# Vitamin D and schizophrenia: 20 years on

**DOI:** 10.1038/s41380-021-01025-0

**Published:** 2021-01-26

**Authors:** Xiaoying Cui, John J. McGrath, Thomas H. J. Burne, Darryl W. Eyles

**Affiliations:** 1 Queensland Brain Institute, University of Queensland, St. Lucia, QLD 4072 Australia; 2 Queensland Centre for Mental Health Research, The Park Centre for Mental Health, Wacol, QLD 4076 Australia; 3 National Centre for Register-Based Research, Aarhus University, 8000 Aarhus, Denmark

**Keywords:** Neuroscience, Physiology

## Abstract

Many epidemiological studies have highlighted the link between vitamin D deficiency and schizophrenia. In particular, two prominent studies report an association between neonatal vitamin D deficiency and an increased risk of schizophrenia. In parallel, much has been learnt about the role of vitamin D in the developing central nervous system over the last two decades. Studies in rodent models of developmental vitamin D (DVD)-deficiency describe how brain development is altered leading to a range of neurobiological and behavioral phenotypes of interest to schizophrenia. While glutamate and gamma aminobutyric acid (GABA) systems have been little investigated in these models, alterations in developing dopamine systems are frequently reported. There have been far more studies reporting patients with schizophrenia have an increased risk of vitamin D deficiency compared to well controls. Here we have conducted a systematic review and meta-analysis that basically confirms this association and extends this to first-episode psychosis. However, patients with schizophrenia also have poorer general health, poorer diets, are frequently less active and also have an increased risk of other medical conditions, all factors which reduce circulating vitamin D levels. Therefore, we would urge caution in any causal interpretation of this association. We also summarize the inconsistent results from existing vitamin D supplementation trials in patients with schizophrenia. In respect to animal models of adult vitamin D deficiency, such exposures produce subtle neurochemical alterations and effects on cognition but do not appear to produce behavioral phenotypes of relevance to schizophrenia. We conclude, the hypothesis that vitamin D deficiency during early life may increase the risk of schizophrenia remains plausible and warrants ongoing research.

## Introduction

Two decades ago, we first hypothesized that low levels of maternal vitamin D increased the risk of schizophrenia [1]. This hypothesis was originally based on “ecological” epidemiology (e.g. season of birth studies), but is now supported by analytical epidemiological studies demonstrating that neonates with vitamin D deficiency have an increased risk of developing schizophrenia later in life [2, 3]. Animal models of developmental vitamin D (DVD)-deficiency were originally created to establish the neurobiological plausibility of this association. A large body of experimental work demonstrates the developmental absence of vitamin D changes the way the brain develops and leads to behaviors in adults that may be of relevance to schizophrenia [4].

The purpose of this review is to summarize the clinical epidemiology literature linking both developmental and adult vitamin D levels with schizophrenia and critically evaluate mechanistic studies in animals that examine the genomic, non-genomic, and epigenetic actions of this neurosteroid in the brain, and in the production of phenotypes relevant to schizophrenia.

### The vitamin D receptor and vitamin D metabolic enzymes are present in the brain

Vitamin D_3_ (cholecalciferol) is produced from 7-dehydrocholesterol in the skin upon ultraviolet B radiation [5]. Cholecalciferol is also present in a small number of foods and can be obtained by supplementation. Cholecalciferol is hydroxylated to 25-hydroxy vitamin D_3_ (25OHD), the major circulating form in the blood, which is then converted into the active hormone, 1,25-dihydroxyvitamin D (1,25(OH)_2_D). 25OHD and 1,25(OH)_2_D can both cross the blood brain barrier [6, 7]. Early immunohistochemistry studies first demonstrated the cellular and subcellular localization of the vitamin D receptor (VDR) and vitamin D metabolizing enzymes in human [8, 9] and rodent brain [8, 10–19].

An exponential growth in high-throughput sequencing technologies has allowed researchers to assess the relative abundance, known isoforms and splice variants of many important genes. As such, the VDR and the major enzymes responsible for the synthesis and metabolism of vitamin D (cytochrome P450 family members CYP27B1 and CYP24A1) in adult human tissues can be accessed using data from the Genotype-tissue expression (GTEx project) [20]. In the GTEx portal, the expression of the VDR is shown in all 11 human brain regions examined including cerebral cortex, cerebellum, amygdala, anterior cingulate cortex, caudate (basal ganglia), hippocampus, hypothalamus, nucleus accumbens (basal ganglia), putamen (basal ganglia), C-1 spinal cord, and pituitary gland. However, the abundance of VDR is relatively low compared to classic VDR organs such as the gut and kidney. Within the brain, the hypothalamus had the greatest abundance. In organs of high VDR abundance anywhere between 3 and 5 isoforms exist whereas the brain contains the single isoform. A number of single nucleotide polymorphism (SNP) variants are associated with VDR’s expression and splicing in tissue such as testis and skin, but not in the brain. Immunohistochemistry studies confirm the VDR is present in developing [9, 17] and adult [12, 15, 16] animal brains with VDR expression in the midbrain being prominently studied [9]. This has recently also been confirmed via single cell sequencing from developing mouse and human midbrains [21].

In respect to vitamin D’s synthetic and metabolic enzymes, the abundance of CYP27B1 is also low in the brain as it is in most other organs apart from kidney. In the brain CYP27B1 levels are most prominent in cerebral cortex and the limbic regions of the caudate putamen and nucleus accumbens. CYP27B1 has seven isoforms with four isoforms detected in kidney and colon, five isoforms in testis, and one in the brain. No correlations between gene variants at the SNP level with enzyme expression or splicing have been reported in the brain. When considering CYP24A1, again expression is low in most organs including brain. The kidney was the only organ shown to contain relatively high levels. CYP24A1 has six isoforms, five of which are present in the kidney, two to four isoforms in other tissue such as bladder and skin, and two isoforms in the brain. Many SNPs are detected in regions likely to be promoter sites indicating they may affect transcription. Indeed such SNPs are associated with CYP24A1 expression in the amygdala, anterior cingulate cortex, cortex, frontal cortex, and nucleus accumbens.

The Allen brain atlas largely confirms that the abundance of VDR and vitamin D-related enzymes is low in human and adult mouse brain and even lower in developing mouse brain [22]. CYP27B1 and CYP24A1 data in the developing mouse brain were not reported in Allen brain atlas.

### Developmental vitamin D deficiency and schizophrenia

Low maternal vitamin D was proposed as a risk factor for schizophrenia almost two decades ago [1]. This hypothesis was prompted by early ecological epidemiological studies that identified that rates of schizophrenia were higher in those: (1) born in winter and spring [23], (2) who live at high latitudes [24], (3) who were raised in urban settings in early life [25–28]. Vitamin D deficiency is a parsimonious candidate that may underlie these observations—as they all correlate with reduced sunlight exposure and thus a higher prevalence of vitamin D deficiency [29]. A separate observation that the offspring of migrants with dark skin who migrate to cold climates have an increased risk of schizophrenia may also be due to low vitamin D during gestation and early life as dark skin requires greater sunlight exposure to make adequate levels of the vitamin D prehormone [30].

These convergent clues led to the hypothesis that maternal vitamin D deficiency may be a risk-modifying factor for schizophrenia. However, schizophrenia is a low prevalence disorder of adult onset. Therefore, in order to test this hypothesis a sample repository that was both sufficiently large and also possessed samples stored for a sufficiently long time to allow a diagnosis of schizophrenia to be made was required. The Danish Biobank Register based on record linkage between the Danish Psychiatric Central Register and the Danish Civil Registration System have collected and stored neonatal blood spots since 1981 making it an ideal sample source [31, 32].

The first Danish case-control study using these neonatal dried blood spots (*N* = 868) found a significant association between low neonatal vitamin D status and subsequent risk of developing schizophrenia (lowest versus reference [fourth] quintile incidence rate ratio (IRR) = 2.1; 95% CI 1.3–3.5) [2]. A second larger Danish case-control study (*N* = 2602) replicated this association between neonatal vitamin D deficiency with an increased risk of schizophrenia (IRR = 1.44, 95% CI: 1.12–1.85) [3].

### Could maternal vitamin D supplementation prevent schizophrenia in offspring?

A Finish birth cohort study found that the absence of vitamin D supplements during the first year of life was associated with an increased risk of schizophrenia in male infants [33]. However, randomized clinical trials to examine the effects of maternal vitamin D supplementation on schizophrenia incidence in adult offspring will, in all likelihood, never be conducted. First, there is no evidence to suggest that increasing 25OHD concentration in those sufficient in vitamin D will influence the health of the offspring (indeed, this strategy may be associated with adverse events). Second, it is not ethical to screen pregnant women for vitamin D deficiency and then randomize this sample to placebo or vitamin D supplementation. Finally, it is not feasible to follow up large mother-offspring samples for 20–30 years in order to examine the risk of schizophrenia. However large-scale maternal supplementation trials with vitamin D are underway for a variety of health outcomes [34] and it will be interesting to see in the future whether such interventions led to a reduction in schizophrenia or more early onset psychiatric disorders such as attention deficit hyperactivity disorder or autism [35–37].

### Vitamin D levels in patients with schizophrenia

Overall, the prevalence of vitamin D deficiency in the general population is high (37.6% based on review of worldwide data [38]). A systematic review and meta-analysis published 6 years ago found that the pooled prevalence of vitamin D deficiency in people with schizophrenia was 65% [39]. A very recent systematic review confirmed an overall 70% prevalence of vitamin D deficiency in people with schizophrenia [40]. We have updated this review (our methods, details of the included studies, forest plots and additional references, and a discussion of the following data can be found in the Supplementary Data). In summary, we identified 43 studies with data related to the association between schizophrenia and vitamin D status (Supplementary Tables 1 and 2). We included 22 studies that provided sufficient data to analyze either standard mean differences (SMD) or odds ratios (OR) in both patients with schizophrenia and controls (Supplementary Fig. 1). With respect to continuous data, based on 15 studies, the SMD was −1.11 (95% CI [−2.03, −0.19], *p* = 0.02). However, high heterogeneity was observed (*I*^2^ = 99.1%, *Q* (df = 14) = 174.04, *p* < 0.0001) (Supplementary Fig. 2). We identified nine studies reporting OR for vitamin D deficiency (at least <50 nmol/l) in patients with schizophrenia. The pooled OR was 2.49 (95% CI [1.17, 5.29], *p* = 0.018), with again high heterogeneity (*I*^2^ = 83.3%, *Q* (df = 8) = 44.06, *p* < 0.0001). A funnel plot showed no publication bias (*p* = 0.72) (Supplementary Fig. 3). As serum 25OHD levels are also known to be associated with latitude [24], we conducted a meta-regression analysis which showed latitude is not a moderator contributing to the heterogeneity of the meta-analysis (Supplementary Fig. 2B, QM(df = 1) = 2.3100, regression coefficient = 0.0208, *p* = 0.1285). This is consistent with a previous meta-analysis [39].

With respect to continuous data from the patients with first-episode psychosis (FEP), based on seven studies, the SMD was (−0.29, 95% CI [−0.65, 0.07], *p* = 0.11). Again, high heterogeneity was observed (*I*^2^ = 75.49%, *Q* (df = 6) = 21.89, *p* < 0.0013) (Supplementary Fig. 4). We identified five studies reporting OR for vitamin D deficiency (at least <50 nmol/l) in FEP patients. The pooled OR was 3.78 (95% CI [2.40, 5.94], *p* < 0.0001). No heterogeneity was detected (*I*^2^ = 0%, *Q* (df = 4) = 3.01, *p* = 0.56) (Supplementary Fig. 5). The findings provide robust evidence confirming earlier smaller meta-analyses [39, 41] that those with schizophrenia have an increased risk of vitamin D deficiency. However this  finding is also consistent with the finding that poor health in general (mental or physical disorders) can lead to vitamin D deficiency mediated by poor diet, reduced physical activity and changes to behavior which are all factors known to increase the risk of vitamin D deficiency [42].

### Vitamin D supplementation in patients with schizophrenia

In keeping with the poor general health status of patients, osteoporosis is highly prevalent (~52%) in patients with schizophrenia [43]. As vitamin D supplementation is the recommended treatment for osteoporosis [44], there exists the opportunity to assess whether vitamin D also affects schizophrenia symptomology. To date, three randomized, double-blind, placebo-controlled clinical trials of vitamin D supplementation either in conjunction with probiotics or maintenance antipsychotic treatment have been conducted. One study showed that vitamin D supplementation (50,000 IU vitamin D/week for 12 weeks) improved positive and negative syndrome scale scores and metabolic profiles including reduction in fasting plasma glucose, and cholesterol levels [45]. The other two clinical trials of vitamin D supplementation (300,000 IU intramuscular injection twice within 3 months [46], or 14,000 IU oral drops per week for 8 weeks) in patients maintaining antipsychotic treatment did not find any significant improvement in symptoms. Other small open-labeled studies also show inconsistent outcomes. One study had shown that 12 months of vitamin D supplementation in schizophrenic patients without hospitalization is associated with lower depressive symptoms and lower rates of current anxiety [47], whereas earlier studies using either 2000 IU orally daily for 8 weeks [48] or low dose dietary (ergocalciferol 400 IU daily for 7 months [49]) did not observe any improvement in symptoms (Supplementary Table 3). So until the results from large-scale, randomized, doubled-blinded placebo-controlled trials intentionally designed to assess the therapeutic effects of vitamin D in patients with schizophrenia such as the DFEND [50] trial are known, at this stage vitamin D supplementation should be recommended for the prevention of osteoporosis, but not the treatment of schizophrenia symptoms.

### Is developmental vitamin D deficiency a biologically plausible risk factor for schizophrenia?

#### DVD deficiency retards brain development in animal models

In line with its pro-differentiation, anti-proliferation, and anti-apoptosis properties, the absence of vitamin D during gestation delays development with more cells proliferating and less being eliminated in the DVD-deficient rat brain [51, 52]. Prolonged cell division also correlated with alterations in the expression of cell cycle-regulating genes including cyclin-dependent kinase p21 and p27 as well as cyclin D1 [51]. Neuroprogenitor cells isolated from ventricular zone of DVD-deficient neonates also exhibit enhanced proliferation [53]. Increased cellular proliferation correlates with abnormal brain structure with larger lateral ventricles and a distortion in brain shape [52]. Consistent with these findings, in CYP27B1 knockout mice (this animal cannot produce the active form of vitamin D) neuronal proliferation is also increased in dentate gyrus [54]. In vitro studies provide further support for the anti-proliferating role of vitamin D in developing brain as application of 1,25(OH)_2_D to rat hippocampal explants inhibited cell division and promoted neurite outgrowth [55]. However, DVD deficiency in the mouse produces a very different developmental brain phenotype with reduced lateral ventricles at embryonic day (E) 17.5 [56, 57]. Whether there are opposite effects on proliferation in the mouse within the DVD-deficient mouse brain remains unknown. However in certain circumstances the addition of vitamin D can also induce proliferation in the brain. Myelination is considered a potential late maturational event that may be abnormal in patients with schizophrenia [58]. When adult rats are chemically demyelinated, oral cholecalciferol supplementation (5000 IU/kg/day) promotes the proliferation and differentiation of neural stem cells in the subventricular zone which migrate into the corpus callosum, differentiate into oligodendrocytes and produce myelin basic protein [59].

The effect of DVD-deficiency on differentiation in rat brain may also be due in part to its known regulation of certain neurotrophic factors critical for neuron maturation [60–65]. DVD deficiency reduces nerve growth factor (NGF), glial-derived neurotrophic factor (GDNF), and the nonselective neurotrophic receptor p75^NTR^ in neonatal rat brains [52, 66]. Brain-derived neurotrophic factor was also reduced in another study but the direction of this finding was dependent on embryonic age [56]. As these trophic factors are well-known to increase neurite outgrowth and dendritic arbor formation [67], it is highly likely their reduction in a DVD-deficient brain may impair early brain connectivity.

#### DVD deficiency alters DA ontogeny

The VDR is prominently expressed in DA neurons within the human substantia nigra [8, 9]. There are many studies of the neuroprotective actions of vitamin D on DA neurons in animal models of Parkinson’s disease [68–77]. Many of the proposed protective actions of vitamin D in adult DA neurons in these studies are also relevant to developing DA neurons. The evidence compiled from the last 20 years supports the hypothesis that vitamin D plays an important role in DA neuron development. The majority of DA neurons are born within the first trimester in a human foetus [78, 79]. In rodents, virtually all DA neurons are born in the first 14 days of gestation which is roughly equivalent to the first trimester in humans [80]. The capacity for vitamin D signaling emerges progressively in the developing mesencephalon along with DA neuron maturation [9]. Between E12 and E15, when the majority of DA neurons are born in rodents [80], DVD deficiency was shown to reduce the expression of DA neuron specification factor Nurr1 and the rate-limiting enzyme in DA synthesis tyrosine hydroxylase (TH) in rat mesencephalon [81, 82]. The N-cadherin signaling pathway could be a possible mediator in the regulation of DA neuron differentiation [83]. During DA neuron maturation, a reduction of TH protein is also observed in substantia nigra of DVD-deficient mouse embryos at E17.5 [56]. In neonatal rats, DVD deficiency alters DA neurotransmitter turnover, consistent with the reduction in related catabolic enzymes such as catechol-O-methyltransferase (COMT) [84–86]. DVD deficiency reduces a major co-receptor for GDNF c-Ret [66]. The effects of vitamin D on all these aspects of DA neuron differentiation and metabolism has been studied in detail in VDR-overexpressing SH-SY5Y neuroblastoma cells and has largely been shown to reverse these deficits. Importantly, the direct genomic regulation of several factors crucial to DA neuron maturation and DA turnover has now been demonstrated in these cells [66, 83, 86]. Alterations in other neurotransmitters have also been reported in DVD-deficient brains such as glutamine, serotonin, and noradrenalin [85].

#### Vitamin D deficiency interacts with well-known epidemiologically validated risk factors for schizophrenia

Epidemiological and translational studies show that prenatal infection increases the risk of developing schizophrenia [87]. Vitamin D is an immune regulator [88]. Therefore, it is interesting to note that placentas from DVD-deficient rat dams produce greater amounts of the inflammatory cytokines IL-6 and IL-1β in response to viral stimulation [89].

Obstetric complications are a well-known risk factor for schizophrenia and the placenta has been considered to be a major mediator for this risk. A recent study revealed that placenta is the central organ mediating the interaction between genetic risk variance and prenatal environmental complications increasing the risk of developing schizophrenia [90]. DVD deficiency in mice also reduces placental weight and expression of genes responsible for placental vascular growth [91].

Glucocorticoids play a critical role in early brain development. Abnormalities in glucocorticoid production in response to maternal stress response have also been closely linked to schizophrenia [92]. DVD deficiency in both rat and mouse increase maternal serum glucocorticoid levels [91, 93]. Consistently, a prolonged vitamin D deficiency during gestation and lactation in rodents alters genes involved in glucocorticoid pathways. For instance 11beta-hydroxysteroid dehydrogenase type II (Hsd 11b2) (a major enzyme that inactivates glucocorticoids) is reduced by DVD deficiency at postnatal day 1 in rat and at embryonic 14.5 in mouse [91, 94]. DVD deficiency also reduces glucocorticoid receptor (mineralocorticoid receptor, nuclear receptor subfamily 3 group C member 2, Nr3c2) expression and increases *Tsc22d* which is a post-receptor mediator of glucocorticoid action in postnatal rat brain and embryonic mouse brain [91, 94].

#### DVD deficiency alters postnatal and adult brain function and behavior

**Fig. 1:**
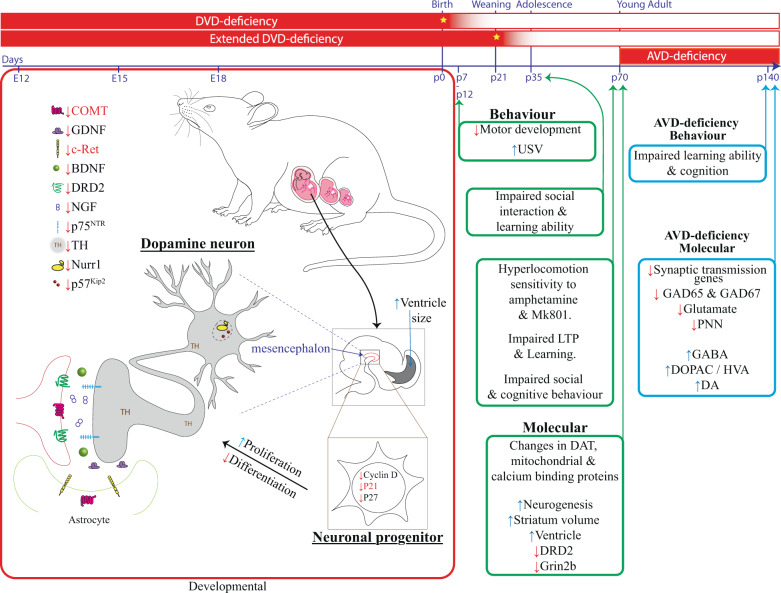
Timelines of vitamin D deficiency in preclinical studies is depicted along with reported alterations. Red boxes depict the period of vitamin D deficiency. Yellow star indicates the time when the normal vitamin D containing diets were reintroduced. Preclinical studies of developmental vitamin D (DVD) deficiency show extensive alterations to developing dopamine (DA) neurons, neuronal differentiation, alterations to brain structure and behavioral phenotypes of interest to schizophrenia (green boxes). DVD deficiency reduces many DA-related genes. Multiple vitamin D response elements (VDREs) have been predicted in the promoters of all genes listed (in silico in black text) using the online software (MoloTool, Transcription Factor Motif Location Toolbox Version 11) [144]. Those genes listed in red have been functionally validated, i.e directly regulated by liganded VDR interacting with VDREs within their promoters. Preclinical studies of adult vitamin D (AVD) deficiency show a number of neurotransmitter alterations and impaired cognition (blue boxes). DVD deficiency developmental vitamin D deficiency, AVD deficiency adult vitamin D deficiency, E12–E18 embryonic days 12–18, p0–140 postnatal days 0–140, COMT Catechol-O-methyltransferase, GDNF glia-derived neurotrophic factor, cRET ret proto-oncogene, DRD2 dopamine receptor D2, NGF nerve growth factor, BDNF brain-derived neurotrophic factor, p75NTR neurotrophin receptor P75, TH tyrosine hydroxylase, Nurr1 nuclear receptor-related 1, p57kip2 cyclin-dependent kinase inhibitor 1C, p21 cyclin-dependent kinase inhibitor 1A, p27 cyclin-dependent kinase inhibitor 1B, USV ultrasonic vocalization, LTP long-term potentiation, DAT dopamine transporter, Grin2b glutamate ionotropic receptor NMDA type subunit 2B, GAD glutamate decarboxylase, PNN perineuronal net, GABA gamma-aminobutyric acid, DOPAC 3,4-Dihydroxyphenylacetic acid, HVA homovanillic acid (Color figure online).

When DVD deficiency is maintained postnatally in rats, offspring have alterations in maternal pup interactions, ultrasonic vocalizations, stereotyped repetitive behavior, delayed motor development, and impaired motor control [94–96]. Adolescent DVD-deficient animals exhibit impaired reciprocal social interaction [95]. As adults, DVD-deficient rats display numerous behavioral phenotypes of relevance to schizophrenia (see summary Fig. 1). As adults, DVD-deficient rats have increased spontaneous locomotion [97], baseline cognitive abnormalities in domains of attention [98, 99], and behavioral sensitivity to psychomimetics such as the NMDA antagonist MK801 [100–102] and the DA releasing agent amphetamine [103]. DVD deficiency also alters hippocampal long-term potentiation (LTP), a neurobiological correlate of learning and memory [104] and impaired learning ability [105]. Whilst displaying some cognitive abnormalities, DVD-deficient mice, however, have far fewer behavioral phenotypes of interest to schizophrenia [57, 106, 107] again suggesting important differences between the species in the role of vitamin D and brain.

With regards to brain structure and function, DVD-deficient adult rats have enlarged lateral ventricles [52, 108] and also altered signaling pathways related to calcium-binding proteins and mitochondrial function [109]. DVD-deficient adult rats also have reduced hippocampal neurogenesis [100, 110]. Interestingly, deficits in LTP, altered neurogenesis and NMDA antagonist induced hyperlocomotion are ameliorated by the antipsychotic haloperidol which blocks dopamine receptors 2 (Drd2) [100, 110] suggesting developmentally induced alterations in DA development may persist into adulthood.

#### DVD deficiency, genomic, and epigenetic mechanisms

Like other neurosteriods, vitamin D acts genomically to regulate transcription but also has faster non-genomic actions. To exert its genomic actions_,_ 1,25(OH)_2_D binds to VDR in the cytoplasm, initiating heterodimerization of the liganded VDR with the retinoic acid X receptor (RXR), which translocates to the nucleus. The liganded VDR/RXR protein complex then binds to vitamin D responsive elements within regulatory regions of target genes. Finally, corepressors are released and coactivators recruited to promote gene expression. In general, the presence of vitamin D stimulates expression of targets [83, 111, 112]. Its absence leads to reduced expression of these same genes in brain [56, 66, 83, 84, 86]. Those genes include COMT and c-Ret proto-oncogene [66, 86, 112], as well as L-type voltage gated calcium channels (L-VGCCs) pore forming unit Cav1.2 (coded by CACNA1C) [113, 114]. Variants in these genes have been frequently linked with schizophrenia [115–117].

VDR’s genomic actions are accompanied by epigenetic processes including histone modification. Histone acetylation is a fundamental step in allowing transcription factor access to DNA. Histones are acetylated by histone acetyltransferase (HAT). Coactivators of the VDR such as p160 or p300/CBP, all have HAT activity to promote transcription in the presence of 1,25(OH)_2_D [118–121]. Conversely, histone deacetylation by histone deacetylase enzymes (HDACs) reverses this process to decrease gene transcription. In the absence of vitamin D, corepressors of VDR either have HDAC activity themselves such as protein ALIEN [122], or interact with HDACs such as the nuclear receptor corepressor and the silencing mediator of retinoid and thyroid hormone receptor to inhibit transcription [123–125].

An interesting recent study from Broekema et al. using a reporter assay show that upon ligand binding to the VDR, 38 corepressors are released and 86 coactivators increase their binding to the VDR [126]. Many of these molecules are shared coregulators for other nuclear receptors and are found in the brain [127]. These coregulator interactions with the VDR are tissue, region, time, and gene specific. The exact co-regulation mechanisms activated or suppressed in the developing or adult brain in the absence of vitamin D remain to be established.

#### Non-genomic mechanisms of vitamin D on the developing brain?

Non-genomic actions occur in time frame of seconds to a few minutes and are obviously independent of transcription. One of the most prominent non-genomic actions of vitamin D is to rapidly enhance L-VGCCs activity in bone, cartilage, cardiac, and skeletal muscle [128–131]. This has only very recently been confirmed in the developing brain. Gooch et al. have now shown 1,25(OH)_2_D rapidly enhances activity-dependent calcium influx via -LVGCC in a small population of neurons in the P10 mouse prefrontal cortex [132]. Considering the aforementioned genomic effects of vitamin D on LVGCC expression in developing neurons [113, 133], we speculate that in the presence of vitamin D deficiency, L-VGCCs properties are transiently altered—this may be a key pathway mediating the impact of DVD deficiency on brain development. Indeed the developmental inactivation LVGCC (deletion of CACNA1C) in mouse forebrain induces behavioral phenotypes of relevance to schizophrenia in adults, including hyperactivity, cognitive impairment, and reduced sociability [134]. Importantly, inactivation L-VGCCs in adult brain in this same study produced no behavioral phenotype of relevance to schizophrenia. The relevance of this potential mechanism to schizophrenia is exemplified by the last major genome-wide association study from the Schizophrenia Working Group of the Psychiatric Genomics Consortium which showed LVGCC subunit variants to be associated with an increased risk for schizophrenia [117].

### The impact of adult vitamin D deficiency on brain function

In contrast to DVD deficiency models where the existing data indicate the development and function of DA systems is primarily affected, AVD deficiency interrupts excitatory and inhibitory neurocircuits and may alter cognitive function. Like DVD deficiency, the impact of AVD deficiency on brain chemistry or behaviors differs across species (rat or mouse).

#### AVD deficiency and neurocircuits

AVD deficiency dysregulates the balance of excitatory and inhibitory neurotransmitters differentially in mice and rats. In BALB/c mice AVD deficiency decreases glutamate and glutamine but increases gamma aminobutyric acid (GABA) and glycine levels [135]. In Wistar rats, vitamin D deficiency enhances baseline glutamate and GABA levels but reduces evoked release of both neurotransmitters. Importantly, vitamin D supplementation rescues the shift between unstimulated and evoked GABA release but only partially restores the impaired glutamate release [136]. The imbalance of excitatory and inhibitory neurotransmitters could be associated with the intensified reactive oxygen species production and elevated free calcium levels in terminals. Another possible mechanism for this dysregulation may be the downregulation of perineuronal nets (PNNs) surrounding inhibitory interneurons in AVD-deficient hippocampus [137]. PNNs are a specialized form of the extracellular matrix that contribute substantially to the excitatory/inhibitory balance by maintaining GABAergic interneuron gamma oscillations [138, 139]. In addition, AVD-deficient mice have a disrupted network centered on the right hippocampus with abnormal connectome within 29 nodes, although there is no direct evidence linking these findings to alterations in PNNs [137]. AVD deficiency also increased GABA levels in Sprague Dawley rats as well as altering striatal DA turnover in rats and mice [135, 140]. In keeping with the difference in behavioral phenotypes between AVD- and DVD deficiency, AVD-deficient mice have a reduced locomotor response to amphetamine [141].

#### AVD deficiency and cognitive function

AVD deficiency induces cognitive impairment in rodent models, though the behavioral outcomes differ between species or strains. AVD deficiency in Sprague Dawley rats increases impulsivity on the 5-choice serial reaction task [140]. AVD-deficient Wistar or Fischer rats also have impairments in hippocampal-dependent memory [142, 143]. AVD-deficient BALB/c mice also have impairments in attention and hippocampal-dependent learning [144].

Overall, there is a growing body of evidence to suggest that AVD deficiency can result in subtle changes in brain neurochemistry and selected behavioral readouts consistent with impaired cognitive function. It is feasible that AVD deficiency may influence the course of preexisting illnesses. For example, an RCT of vitamin D supplementation in patients with Parkinson’s disease found the expected decline over time in the placebo group (in keeping with progression of this neurodegenerative disorder). In contrast, the active vitamin D group preserved neurological function [145]. Thus, the absence of the expected concentration of 1,25(OH)_2_D may accelerate progression of a prior disorder [146]. It remains to be seen if these factors also influence recovery or progression of psychiatric disorders.

### What has the last 20 years research produced?

The last 20 years has seen neonatal vitamin D deficiency emerge as a plausible candidate risk factor for schizophrenia. Earlier studies examining environmental phenomena such as season of birth as proxy markers for DVD deficiency have given way to direct analytical epidemiological studies [2, 3]. Such studies now await replication in other tissue repositories. The last 20 years have produced substantial preclinical work establishing vitamin D as an essential neurodevelopmental steroid. Preclinical studies have provided robust evidence that DVD deficiency adversely affects brain structure, delays brain cell maturation, and produces neurochemical and behavioral phenotypes of relevance to schizophrenia. In particular, there has been a concerted effort in preclinical studies suggesting that alterations in vitamin D status affect the ontogeny and survival of DA neurons. Altered DA neuron ontogeny is emerging as a plausible convergent mechanism in broader risk factor epidemiology in schizophrenia [147]. Of relevance here are recent studies showing a systemic injection of 1,25(OH)_2_D in pregnant animals at early developmental stages not only blocked maternal inflammation induced behavioral phenotypes related to subcortical hyperdopaminergia but also normalized early aspects of DA neuron gene expression and early DA neuron migration [111, 148].

With respect to vitamin D levels in patients, systematic reviews have convincingly shown that people with schizophrenia are at increased risk of vitamin D deficiency. Whilst there is no convincing evidence that low vitamin D levels adversely affects adult brain health from a clinical perspective, vitamin D deficiency in this patient group should still be taken seriously. Low vitamin D levels are also associated with general physical health problems, including adverse cardiovascular outcomes [149] and so may potentially compound the poor health status associated with psychotic disorders and their treatment [150]. This issue should therefore be incorporated in general dietary advice for patients with schizophrenia.

In preclinical studies, AVD deficiency appears to have a subtle but consistent impact on learning and memory. Although there is some evidence that AVD deficiency induces alterations in the balance of excitatory/inhibitory neurotransmitters [135] and/or in PNN density [137] any underlying mechanism linking AVD deficiency and altered cognition remains elusive. It is feasible that vitamin D deficiency may contribute to poorer clinical outcomes in those with preexisting brain disorders [151]. Thus, ongoing research into these mechanisms are warranted.

### Future directions

#### Is there a critical window of vitamin D deficiency during development?

The existing evidence base connecting DVD deficiency and schizophrenia is limited to newborns. However, it is feasible that low serum vitamin D during childhood and puberty could also affect brain development. For instance, absence of vitamin D supplementation during the first year of life also correlates with increased incidence of schizophrenia [33]. Additionally, dark skinned (people with dark skin require greater sunlight exposure to make adequate levels of vitamin D [152]) migrants to the Netherlands (children who immigrated at age 3–17 years) have increased risk of later psychotic disorders [30]. This suggests that postnatal vitamin D deficiency may also adversely affect brain development. This hypothesis is supported by limited evidence from preclinical studies. When vitamin D deficiency is extended to weaning (postnatal day 21, extended DVD deficiency), the ventriculomegaly seen in DVD-deficient neonates persists to adulthood [108]. Vitamin D deficiency during puberty in a rat also decreases glutamate and GABA uptake by reducing the expression of glutamate (EAAC-1) and GABA (GAT-3) transporters [153]. However, a recent study purposely designed to examine negative and cognitive symptom phenotypes in response to varying pre- and postnatal windows of vitamin D deficiency failed to show any exacerbation of behavioral phenotypes with prolonged DVD deficiency. Instead, all pre- and postnatal windows of DVD deficiency impaired novel object recognition [154]. Clearly, further preclinical studies are needed to examine the effects of postnatal vitamin D deficiency on brain structure and function. Until then, the importance of postnatal vitamin D deficiency to schizophrenia risk remains unknown.

#### How does DVD deficiency affect gene regulation in developing brains?

In terms of understanding vitamin D’s role in gene regulation in developing and adult brains, there is still much we do not understand. Some studies have provided evidence for a direct genomic interaction between the liganded VDR with promoters of known DA-related gene targets in developing brains [66, 86]. However, it is also highly likely that vitamin D’s well-described epigenetic control of transcription in cancer cells via histone acetylation, promoter methylation or via miRNAs is also prominent in brain though this remains virtually unexplored. Given clear and consistent effects of DVD- and AVD deficiency on the expression of certain genes highly relevant to brain development and function such research should now be conducted.

## Conclusion

Neonatal vitamin D deficiency is associated with an increased risk of schizophrenia. The ease of manipulating this dietary factor has facilitated a vast amount of preclinical studies in rat and mouse both in developing and in adult animals. Animal models were initially examined to establish the biological plausibility of this risk relationship. However, the real power of such studies is their ability to uncover potentially vulnerable or disease causative pathways. Whilst there are likely to be many upstream causative agents and pathways to schizophrenia the consistent findings from DVD deficiency showing abnormalities in how DA neurons develop and function in adult brains and the links emerging between GABA neurons and cognitive deficits in AVD-deficient animals is tantalizing as these are highly plausible disease causative pathways (see summary Fig. 1). Adequate vitamin D levels have long been considered essential for bone health. The last 20 years of data have revealed that vitamin D deficiency also impacts on brain development and function.

## Supplementary information

supplementary meta analysis

Supplementary Tables
