# Supplementary material for: Vitamin D and schizophrenia: 20 years on
Source: Mol Psychiatry. 2021 Jan 26;26(7):2708–20. doi: 10.1038/s41380-021-01025-0 (PMC8505257; doi:10.1038/s41380-021-01025-0)
Supplement: Supplementary file 1 — supplementary meta analysis [file 41380_2021_1025_MOESM1_ESM.pdf]

Method of literature selection

A systematic search was conducted using MEDLINE (Pubmed) on August 10th 2020. The following combination of key words was used: (schizophrenia OR psychosis OR psychiatric disorders) AND (cholecalciferol OR vitamin D OR 25-hydroxyvitamin D_2 OR 25-hydroxyvitamin D_3 OR 1 alpha, 25 hydroxyvitamin D_3). We retrieved 2579 references from MEDLINE and two from snowball searching [1,2] . The literature were selected if they were: 1) written in English; 2) an epidemiological research article in human population (case report, preclinical studies, reviews, and comments were excluded); 3) providing data that could be used to calculate standard mean difference (SMD), and estimate the odds ratio (OR) and corresponding 95% confidence intervals (CIs). A total of 43 full articles were included in supplementary tables 1 and 2. Twenty-two studies were included in the systematic review and meta-analysis. 15 studies were used to compare standard mean difference in 25OHD levels between chronic schizophrenia patients and healthy controls. Nine studies were used for meta-analysis of vitamin D deficiency and the risk of schizophrenia (six of these studies overlap with suppl Fig 2, three additional studies from suppl Table 1 contained mean 25OHD values only, therefore were not used for SMD however these three studies contained the prevalence of vitamin D deficiency, so were included for OR meta-analysis). Seven studies were used for meta-analysis of SMD in first episode psychosis (FEP). Four studies report 25OHD levels in FEP only. Three additional studies had FEP patients within a larger schizophrenia cohort (from suppl table 1). Five studies were used to estimate pooled OR in FEP, three of these five studies overlap with suppl Fig 4. Two additional studies from suppl Table 1contained mean 25OHD values only, therefore could not be used for SMD, however this study contained the prevalence of vitamin D deficiency, so was included for OR meta-analysis.

All meta-analysis was conducted using the '*Metafor*' package in R (version 3.6.3) [3]. Because our prior expectation was that the effect sizes between the variables of interest would vary between studies, the pooled association (SMD, OR) was estimated using the random effects model. ORs from each study were transformed by taking the natural logarithm and standard errors were back-calculated from the reported confidence intervals. ORs were chosen as the effect size measure because they were reported in most of the included studies. Heterogeneity between studies was assessed using the Q-statistic and I^2. The potential for

publication bias was explored using funnel plots. The weights of each study were calculated using inverse-variance weighting method. The distribution of the estimates were tested using the Egger test. I^2 values at 0-25%, 25-50%, 50-75%, 75%-100% represent very low, moderate, substantial and high heterogeneity, respectively.

Supplementary Figure 1. Flow chart of literature selection

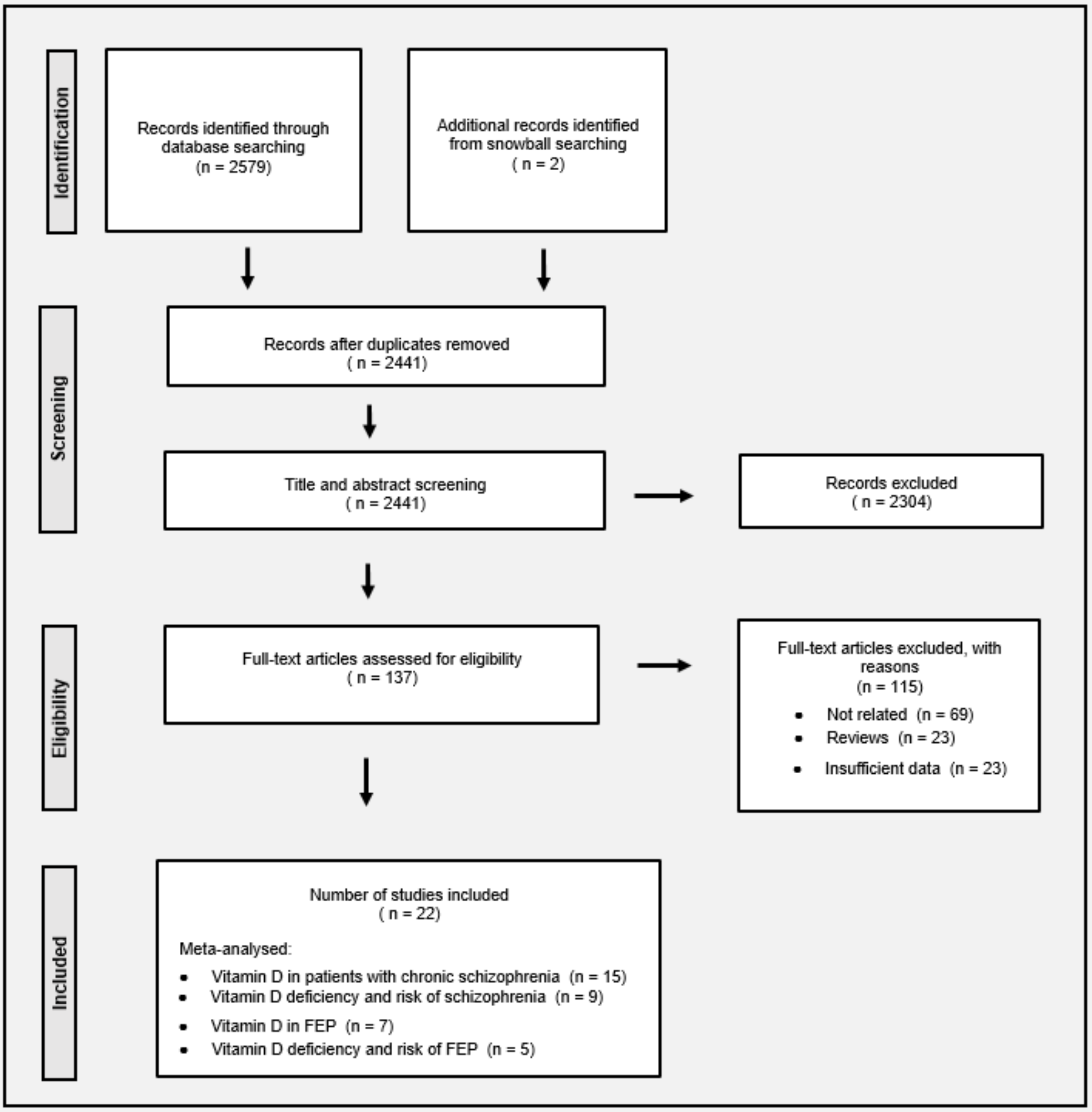

Supplementary Figure 2 Forest plot of the standard mean difference (SMD) of vitamin D in schizophrenia patients compared to healthy controls.

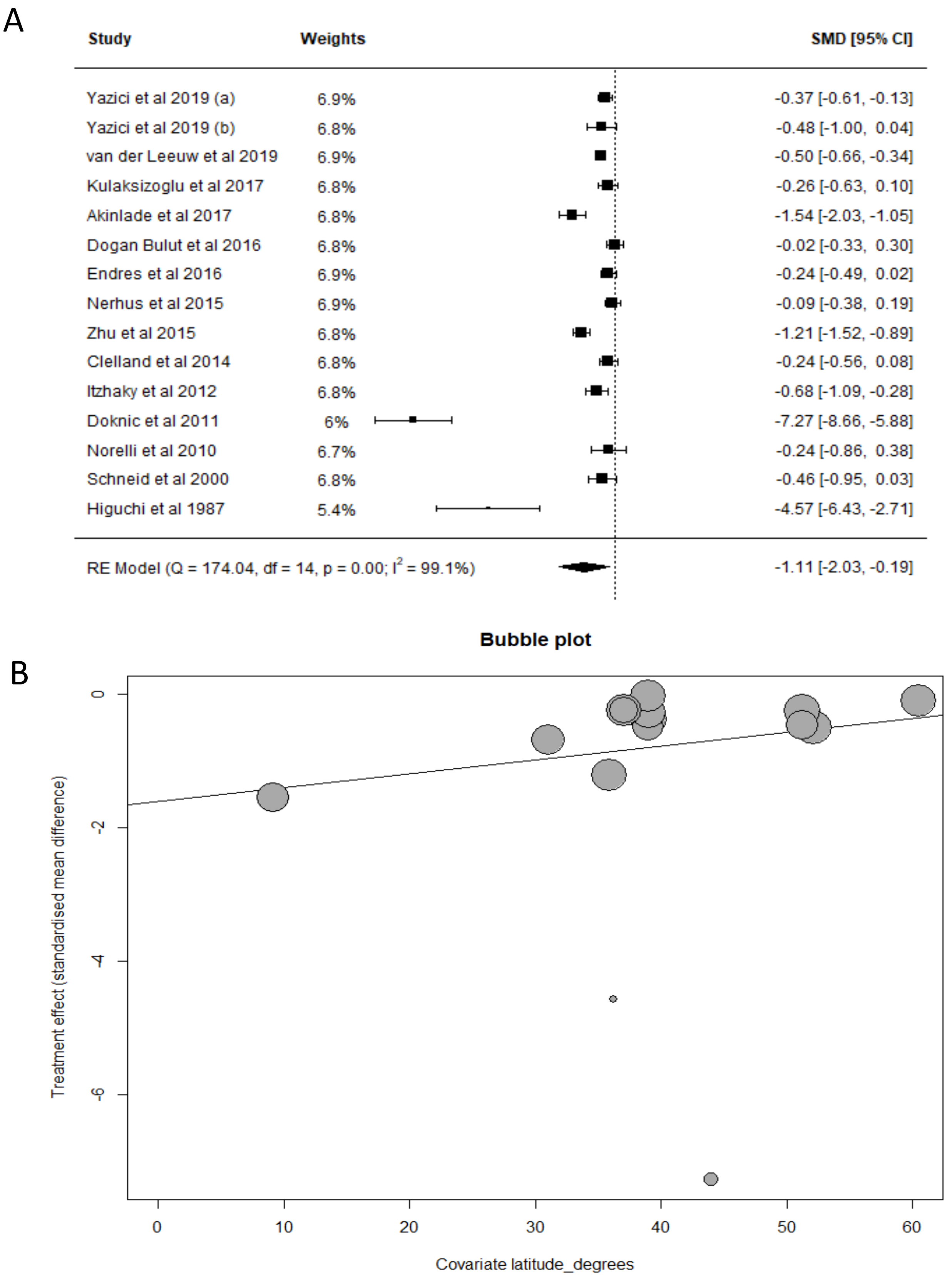

Supplementary Figure 2. The standard mean difference (SMD) of vitamin D levels between patients with schizophrenia and healthy individuals. (A) Black squares indicate the SMD in each study, with square sizes inversely proportional to the standard error of the SMD. Horizontal bars represent 95% CI. The summary estimate (black diamond) was obtained by using a random-effects model. A significant difference in 25OHD serum levels was found in the schizophrenia patients compared to the control population (SMD = -1.11, 95%CI [-2.03, -0.19], $p = 0.02$). However high heterogeneity was observed ($I^2 = 99.1\%$, Q (df = 14) = 174.04, $p < 0.0001$). (B) Bubble plot for meta-regression of correlation between SMD and latitude of the studies. Each bubble depicts a separate study (15 studies) and the bubble size is inversely proportional to the variance of SMD. No significant correlation was detected (QM(df = 1) = 2.3100, regression coefficient = 0.0208, $p = 0.1285$).

Supplementary Figure 3. Forest plot of the Odds Ratio of vitamin D deficiency in schizophrenia patients vs. healthy controls

A

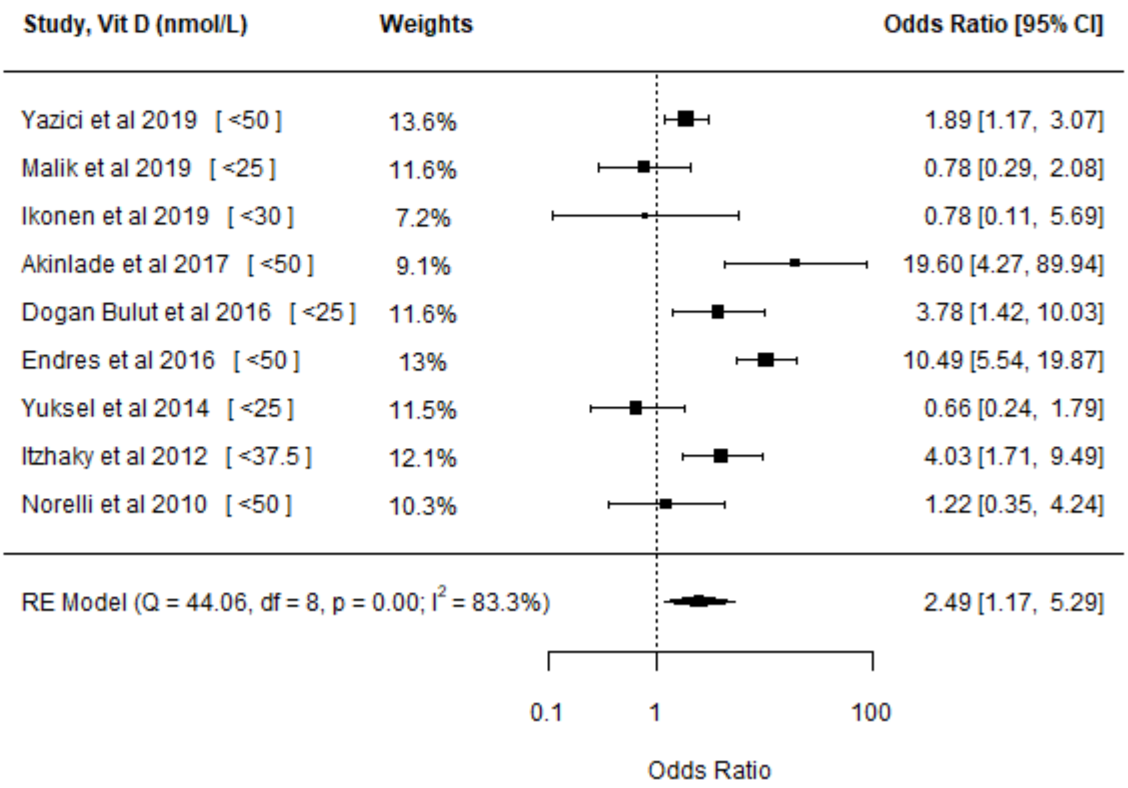

B

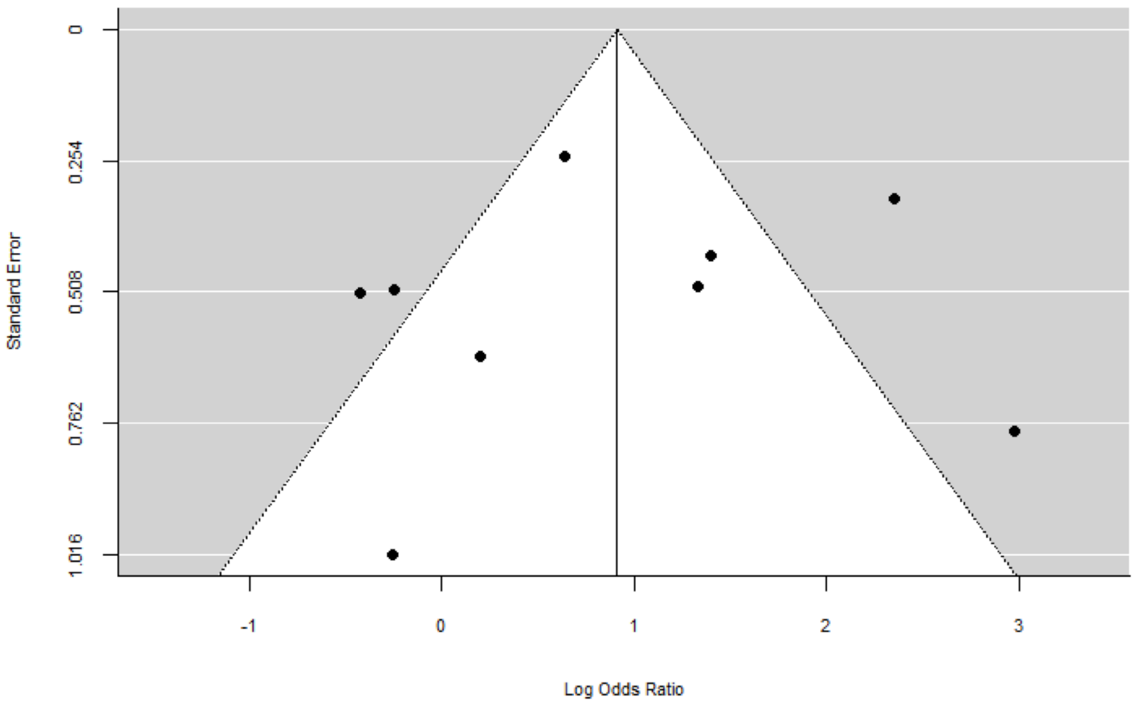

Supplementary Figure 3. Forest plot of Odds Ratios (OR) of vitamin D deficiency (based on different thresholds reported from individual studies) in schizophrenia patients vs. healthy

controls and Funnel plot. (**A**) Black squares indicate the OR in each study, with square sizes inversely proportional to the standard error of the OR. Horizontal bars represent 95% CI. The summary estimate (black diamond) was obtained by using a random-effects model. Meta-analysis identified an inverse association between vitamin D deficiency and schizophrenia. The pooled OR was 2.49 (95% CI [1.17, 5.29], $p = 0.018$), with again high heterogeneity (I^2=83.3%, Q (df = 8) = 44.06, $p < 0.0001$). (**B**) Funnel plot showed no publication bias (p = 0.72).

Supplementary Figure 4. Forest plot of the standard mean difference (SMD) of 25OHD levels in first episode psychosis (FEP) patients compared to healthy controls.

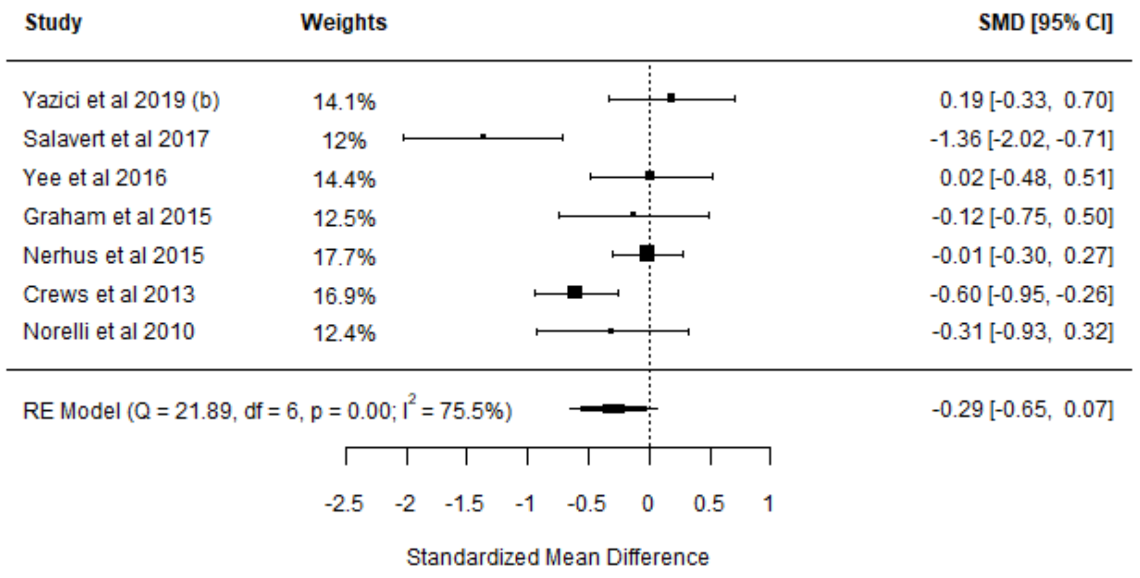

Supplementary Figure 4. The standard mean difference (SMD) of vitamin D levels between first episode psychosis (FEP) patients and healthy individuals. Black squares indicate the SMD in each study, with square sizes inversely proportional to the standard error of the SMD. Horizontal bars represent 95% CI. The summary estimate (black diamond) was obtained by using a random-effects model. No significant difference in the serum levels of 25OHD in FEP was found when compared to the control population (SMD = -0.29, 95%CI [-0.65, 0.07], $p = 0.11$). Again high heterogeneity was observed ($I^2 = 75.5\%$, Q (df = 6) = 21.89, $p < 0.0001$).

Supplementary Figure 5. Forest plot of the Odds Ratio of vitamin D deficiency in FEP vs. healthy controls.

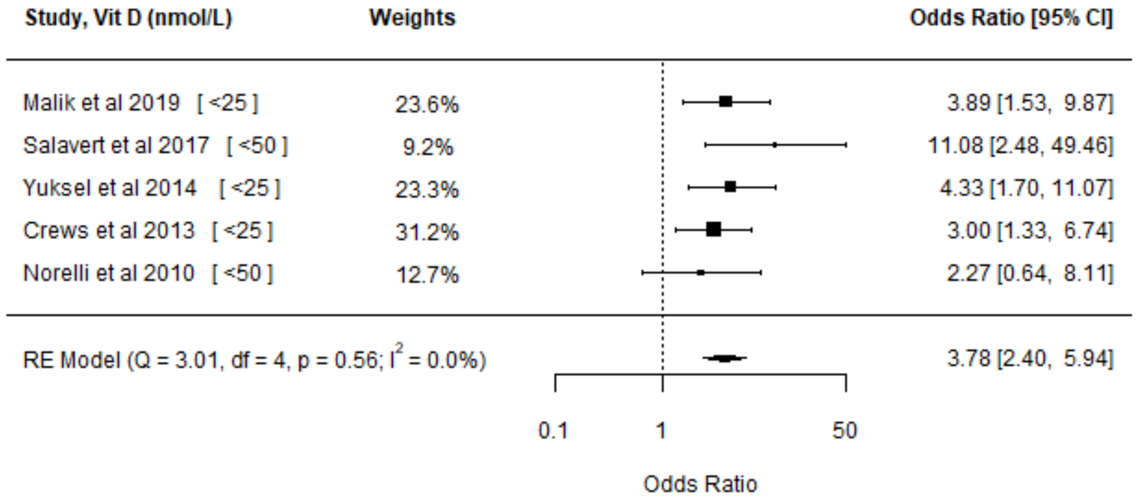

Supplementary Figure 5. Forest plot of the Odds Ratio (OR) of vitamin D deficiency (based on different thresholds reported from individual studies) in first episode psychosis (FEP) vs. healthy controls. Black squares indicate the odds ratio OR in each study, with square sizes inversely proportional to the standard error of the OR. Horizontal bars represent 95% CI. The summary estimates (black diamond) were obtained by using a random-effects model. The pooled OR of vitamin D deficiency was 3.78 (95% CI [2.40, 5.94], $p < 0.0001$). No significant heterogeneity was detected ($I^2 = 0\%$, Q (df = 4) = 3.01, $p = 0.56$)

Discussion

Although our analysis would appear to provide some support for the link between inadequate levels of vitamin D in patients with schizophrenia, we would urge caution in the interpretation of this association for two main reasons. People with schizophrenia have poorer general health, poorer diets and are frequently less active. Patients with schizophrenia also have an increased risk of a subsequent medical condition [4]. All of these factors are also known to reduce 25OHD levels. In addition, a recent large genome wide association study of 25OHD levels has provided new insights into the factors that influence 25OHD concentrations [5]. This study found no evidence that common genetic variants associated with 25OHD mediated an increased risk of schizophrenia. However, common variants associated with schizophrenia (and a wide range of other mental disorders) and vulnerability to chronic disorders in general were associated with lower 25OHD concentrations. The authors of this article speculate that vulnerability to chronic disorders leads to reduced outdoor activity, and thus an increased risk of vitamin D deficiency. Therefore, it is highly likely that a person with a chronic condition such as schizophrenia will have lower vitamin D levels than the general population thus confounding any causative link. We conclude that until large well-designed studies that control for the general health and activity of the individual are conducted any consideration the vitamin D status in an adult contributes to schizophrenia symptoms must be considered at best preliminary.

References

1 Malik BR, Zafar MH & Khan S. Role of Vitamin D as adjuvant therapy of hypovitaminosis D in patients of schizophrenia. *Pakistan Journal of Medical and Health Sciences* 2019; **13**: 303-305.

2 Norelli L, Coates A & Kovasznay B. A comparison of 25-hydroxyvitamin D serum levels in acute and long-stay psychiatric inpatients: A preliminary investigation. *E Spen Eur. E J. Clin. Nutr. Metab.* 2010: e187-e189.

3 Balduzzi S, Rucker G & Schwarzer G. How to perform a meta-analysis with R: a practical tutorial. *Evidence-based mental health* 2019; **22**: 153-160.

4 Momen NC, Plana-Ripoll O, Agerbo E, Benros ME, Borglum AD, Christensen MK *et al.* Association between Mental Disorders and Subsequent Medical Conditions. *N. Engl. J. Med.* 2020; **382**: 1721-1731.

5 Revez JA, Lin T, Qiao Z, Xue A, Holtz Y, Zhu Z *et al.* Genome-wide association study identifies 143 loci associated with 25 hydroxyvitamin D concentration. *Nat Commun* 2020; **11**: 1647.
