# Supplementary material for: Vitamin D and schizophrenia: 20 years on
Source: Mol Psychiatry. 2021 Jan 26;26(7):2708–20. doi: 10.1038/s41380-021-01025-0 (PMC8505257; doi:10.1038/s41380-021-01025-0)
Supplement: Supplementary file 2 — Supplementary Tables [file 41380_2021_1025_MOESM2_ESM.pdf]

Supplementary Table 1 Serum vitamin D levels in patients with chronic schizophrenia.

Studies	Subjects<br>nSZ(nMale)<br>nCtrl(nMale)	Mean age (Year)	25OHD (nM) Mean ± SD		Definition of deficiency and insufficiency (nM) and prevalence (%)			In/Out Patient	Country	Outcomes (Association between vitamin D levels and symptoms, biochemistry and brain structure in SZ patients)
			SZ	Ctrl	nM	SZ	Ctrl			
Yazici et al. 2019a [1]	189(108)<br>109(59)	41.4	43.8 ± 24.1	53.2 ± 27.3	< 50<br>< 75	66.5%<br>21.9%	51.5%<br>35.0%	In	Turkey	N/A
Yazici et al. 2019b [2]	30(30)<br>28(28)	40.5	28.4 ± 11.5	34.2 ± 12.3				Out	Turkey	No correlation between serum vitamin D levels and PANSS scores
Van der Leeuw et al. 2019 [3]	347(268)<br>282(118)	30.3	47.0 ± 26.1**	60.1 ± 26.5	< 75	82.8%	62.5%	Both	Netherland	An inverse association between serum vitamin D levels and positive and negative symptom scores
Malik et al. 2019 [4]	40(26)<br>40(18)	40.0	37.6	37.6	< 25<br>< 50	25.0%<br>67.5%	30.0%<br>50.0%	Out	Pakistan	N/A
Peitl et al. 2019 [5]	97(45)	40.2	53.6 ± 29.0					In	Croatia	A positive association between serum vitamin D levels and neuregulin 1
Arranz et al. 2019 [6]	199(103)	47	42.0		< 50	73.7%		Out	Spain	An inverse association between serum vitamin D and the risk of presenting metabolic syndrome
Zoghbi et al. 2019 [7]	196(118)	37.3			< 50	46.5%		In	Lebanon	Severe vitamin D deficiency was associated with an increase in MRSS score
Fond et al. 2019 [8]	251(180)	35.4			< 25	27.5%		Out	France	An inverse association between serum vitamin D levels and negative symptoms, high suicide risk, anti-depressant consumption.
Ikonen et al. 2019 [9]	40(22)<br>4659(2315)	31	65.5	74.3	< 30<br>< 50	2.5%<br>27.5%	3.2%<br>25.2%	Out	Northern Finland	N/A
Coentre et al. 2019 [10]	33(23)	41.2	40.5 ± 28.2					Out	Portugal	An inverse association between serum vitamin D levels and general psychopathology
Gurholt et al. 2018 [11]	90 (43)<br>91(54)	30.6	48.8 ± 17.6	53.5 ± 20.0				Both	Norway	A positive association between serum vitamin D levels and intracranial volume
Patel et al. 2018 [12]	18	40.6	29.5 ± 10.3					In	UK	N/A
Bruins et al. 2018 [13]	1840(1174)	44.7	48.5 ± 27.4		< 50	67.2%		Out	Netherland	Logarithmic serum vitamin D levels was association with the logarithmic PANSS Negative Subscale
Kulaksizoglu et al. 2017 [14]	64(36)<br>54(31)	38.3	25.2 ± 6.6	27.2 ± 8.6				Out	Turkey	A negative correlation between serum vitamin D levels and PANSS-negative symptom score, and triglyceride and insulin levels. A positive correlation between vitamin D levels and HDL levels

Akinlade et al. 2017 [15]	60<br>30	35.1	49.4 ± 13.0***	70.2 ± 14.1	< 50<br>< 75	58.3%<br>40.0%	6.7%<br>56.7%	In	Nigeria	No correlation between serum vitamin D levels and PANSS scores
Dogan Bulut et al. 2016 [16]	80(42)<br>74(39)	36.6	58.7 ± 35.0	59.2 ± 24.0	< 25<br>< 50	25.0%<br>13.8%	8.1%<br>28.4%	Out	Turkey	An inverse association between serum vitamin D levels and the severity of positive and negative symptoms
Endres et al. 2016 [17]	60(35)<br>3917(1706)	33.5	37.5 ± 24.5	45.0 ± 31.5	< 50<br>< 75	80.0%<br>15.0%	57.3%<br>20.8%	In	Germany	N/A
Lally et al. 2016 [18]	324(193)	43.6	31.0 ± 18.3		< 25<br>< 50	48.8%<br>37.3%		Out	England	An inverse association between serum vitamin D levels and cardiovascular disease risk factors and metabolic syndrome
Bazzano et al. 2016 [19]	61	44	54.5 ± 29.9		< 50<br>< 75	51.4%<br>35.1%		In	USA	N/A
Bogers et al. 2016 [20]	28(24)<br>29(10)	44	33<br>median	73<br>mediam	<50	79%		In	Netherlands	N/A
Shivakumar et al. 2015 [21]	35(20)	32.1	36.3 ± 14.3		< 50<br>< 75	83.0%<br>14.0%		Out	India	A positive association between serum vitamin D levels and right hippocampal grey matter
Nerhus et al. 2015 [22]	71(46)<br>142(92)	28.0	43.0 ± 20.3	45.0 ± 21.4				Both	Norway	No correlation between serum vitamin D levels and PANSS scores
Zhu et al. 2015 [23]	93(41)<br>93(52)	29.5	26.4 ± 13.3***	43.7 ± 15.2				Out	China	An inverse association between serum vitamin D levels and CRP
Yuksel et al. 2014[24]	41(27)<br>40(19)	38.9	37.6<br>(median)	37.6<br>(median)	< 25<br>< 50	22.0%<br>68.3%	30.0%<br>55.0%	Out	Turkey	A negative correlation between serum vitamin D levels and CGI and PANSS scores
Clelland et al. 2014 [25]	64(31)<br>90(46)	38.5	79.1 ± 56.6	92.7 ± 56.8	< 75	68.8%	46.7%	In	USA	N/A
Cieslak et al. 2014 [26]	22(13)	44.3	43.3 ± 22.3		< 75	90.9%		Both	USA	A strong inverse association between low serum vitamin D and negative symptoms in males
Itzhaky et al. 2012 [27]	50(34)<br>50(13)	40.2	37.5 ± 18.3*	50.5 ± 19.5	<37.5<br>< 75	57.1%<br>40.8%	24.5%<br>63.3%	In	Israel	No correlation between serum vitamin D levels and PANSS scores
McCue et al. 2012 [28]	107(70)	43.3	52.8 ± 26.0		< 50	52.3%		In	North America	No relationship between serum vitamin D levels and major psychiatric diagnosis categories
Abdullah et al. 2012[29]	185	40.0	52.5		< 80	88.6%		In	USA	No association between serum vitamin D levels and BPRS
Menkes et al. 2012 [30]	49		34.5 ± 15.4		< 25<br>< 50	26.5%<br>59.2%		In	New Zealand	N/A
Murie et al. 2012 [31]	25	47.0			< 25<br>< 50	56%<br>36%		In	UK	N/A
Doknic et al. 2011 [32]	26(12)<br>35(11)	31.3	23.6 ± 2.8<br>***	71.9 ± 8.3				Out	Serbia	N/A

Partti et al. 2010 [33]	48(20)<br>6100(2808)	53.5	38.9	45.1				Out	Finland	N/A
Humble et al. 2010 [34]	20(8)	47.4	35 (median)					Out	USA	N/A
Norelli et al. 2010 [35]	20(15)<br>20(11)	46.1	50.0 ± 22.3	56.8 ±32.5	< 50<br>< 75	50.0%<br>30.0%	45.0%<br>35.0%	In	USA	N/A
Rey-Sanchez et al. 2009 [36]	73(48)<br>73(48)	F59.8<br>M61.9	F 51.1 ± 65.1 ***<br>M 37.8±29.9	F 82.8±48.6<br>M 45.3±38.6	<37.5	F 74.1%<br>M 69.6%		In	Spain	An inverse association between serum vitamin D levels and hyperparathyroidism, hyperprolactinaemia and accelerated bone turnover in female patients.
Bergemann et al. 2008 [37]	72(0)<br>71(0)	33.8	40.8 ± 19.8 ***	61.5 ± 28.8				In	Germany	N/A
Schneider et al. 2000 [38]	34(19)<br>31(19)	38.9	87.8 ± 65.3*	114.8.± 49.5				Out	Germany	N/A
Higuchi et al. 1987 [39]	12(5)<br>5(5)	35.9	31.5 ± 5.5**	55.8 ± 3.5				In	Japan	N/A

Where indicated serum 25OHD levels were significantly lower in schizophrenia patients compared to the heathy controls (*p < 0.05, **p < 0.01 ***, p <0.001). Abbreviations in the Table: SZ, schizophrenia; Ctrl, control; CRP, C-reactive protein; PANSS, the Positive and Negative Syndrome Scale; CGI, Clinical Global Impression; MRSS, Morningside Rehabilitation Status Scale; HDL, high density lipoprotein; BPRS, The Brief Psychiatric Rating Scale. N/A indicates that the study did not examine the association between serum 25OHD levels and psychotic symptoms/scores, or other biochemical and brain structural outcome.

Supplementary Table 2. Vitamin D deficiency and First Episode Psychosis (FEP)

Studies	Subjects nFEP(nMale) nCtrl(nMale)	Mean Age (Year)	25OHD (nM) Mean ± SD		Definition of vitamin D deficiency or insufficiency (nM) and prevalence (%)			In/Out Patient	Country	Outcomes (Association between vitamin D levels and symptoms, biochemistry and brain structure in FEP patients)
			FEP	Ctrl	(nM)	FEP (%)	Ctrl (%)			
Yazici et al. (b) 2019 [2]	30(30)<br>28(28)	40.6	40.5 ± 44.8	34.2 ± 12.3				In	Turkey	No correlation between serum vitamin D levels and PANSS scores
Malik et al. 2019 [4]	40(26)<br>40(18)	38.7	17.9***	37.5	< 25<br>< 50	63.5%<br>30.0%	30.0%<br>50.0%	Out	Pakistan	N/A
Coentre et al. 2019 [10]	33(23)	31.2	45.4 ± 18.7					In	Portugal	An inverse correlation between vitamin D levels and the severities of depressive and negative symptoms and general psychopathology
Lally et al. 2019 [40]	168(108)	29.3	34.5 ± 23.3		< 50	80.0%		Out	UK	A negative association between baseline serum vitamin D levels and negative psychotic symptoms at one year after FEP.
Salavert et al. 2017[41]	22(16)<br>22(6)	31.1	32.9 ± 14.9 **	56.6 ± 19.1	< 50<br><75	86.4%<br>13.6%	36.4%<br>40.9%	In	Spain	N/A
Yee et al. 2016 [42]	31(15)<br>31(14)	29.1	61.7 ± 26.7	61.3 ± 22.6				In	Singapore	A negative association between serum vitamin D levels and negative symptoms
Graham et al. 2015 [43]	20(12)<br>20(12)	23	70.5 ± 31.5	74.8 ± 35.8	< 75	55.0%		Out	USA	A reverse correlation between serum vitamin D levels and negative and cognitive deficits.
Nerhus et al. 2015 [22]	71(46)<br>142(92)	27.3	44.8 ± 19.5	45.1 ± 21.4				Both	Norway	N/A
Yuksel et al. 2014 [24]	40(19)<br>40(20)	38.1	17.9 (median)	37.6 (median)	< 25<br>< 50	65.0%<br>30.0%	30.0%<br>55.0%	In	Turkey	An inverse association between serum vitamin D levels and negative symptoms
Crews et al. 2013 [44]	69(27)<br>69(27)	31	36.5 ± 23.0***	53.8 ± 33.0	< 25<br>< 50	36.2%<br>39.0%	15.9%<br>43.0%	In	UK	N/A
Dealberto et al.2013 [45]	20(11)		38.0 ± 10					In	Canada	N/A
Norelli et al. 2010 [35]	20(17)<br>20(11)	39.4	47.8 ± 24.5	56.8 ± 32.5	< 50<br>< 75	65.0%<br>20.0%	45.0%<br>35.0%	In	USA	N/A

Where indicated serum 25OHD levels were significantly lower in FEP compared to the heathy controls (Ctrl) (**$p < 0.01$ ***, $p < 0.001$). N/A indicates that the study did not examine the association between serum vitamin D levels and psychotic symptoms/scores, or other biochemical and brain structural outcomes.

Supplementary Table 3. Clinical trials of vitamin D supplementation in schizophrenia

Studies	Vitamin D nSupplementation(nMale) nPlacebo (nMale)	Age (Year)	country	Illness Length (Year)	Vitamin D supplementation (dose and duration)	Outcomes (Association between vitamin D supplementation and symptoms and biochemistry in SZ patients)
Ghaderi et al. 2019 [46]	30(28) 30(28)	44.8	Iran	2	50,000 IU every 2 week for 12 weeks plus probiotic	Improved the general and total PANSS scores and metabolic profiles
Fond et al. 2018 [47]	12(8) 30(25)	36	France	16.4	12 months	Improved depressive symptoms and lower rates of current anxiety disorder.
Krivoy et al. 2017[48]	24(18) 23(14)	39.4	Israel	16	14,000IU per week for eight weeks (maintaining clozapine)	A trend to improve cognition
Sheikhmoonesi et al. 2016[49]	40(40) 40(40)	46.2	Iran	6.3	Two intramuscular vitamin D injection at 300 000IU in three month	No improvement
DealBerto et al. 2016 [45]	8 9		Canada		1000IU vitamin D	No improvement
Thakurathi et al. 2013 [50]	19(12)	50.2	USA	4.9	2000IU daily for 8 weeks	Reducing total cholesterol
Tiangga et al. 2008 [51]	17(17)		UK	>1year	Calcium and egocalciferol (400U) for 7 month	Increasing 25OHD levels

References

1 Yazici AB, Akcay Ciner O, Yazici E, Cilli AS, Dogan B & Erol A. Comparison of vitamin B12, vitamin D and folic acid blood levels in patients with schizophrenia, drug addiction and controls. *J. Clin. Neurosci.* 2019; **65**: 11-16.

2 Yazici E, Mutu Pek T, Guzel D, Yazici AB, Akcay Ciner O & Erol A. Klotho, vitamin D and homocysteine levels during acute episode and remission periods in schizophrenia patients. *Nord J Psychiatry* 2019; **73**: 178-184.

3 van der Leeuw C, de Witte LD, Stellinga A, van der Ley C, Bruggeman R, Kahn RS *et al.* Vitamin D concentration and psychotic disorder: associations with disease status, clinical variables and urbanicity. *Psychol. Med.* 2019; **50**: 1680-1686.

4 Malik BR, Zafar MH & Khan S. Role of Vitamin D as adjuvant therapy of hypovitaminosis D in patients of schizophrenia. *Pakistan Journal of Medical and Health Sciences* 2019; **13**: 303-305.

5 Peitl V, Silic A, Orlovic I, Vidrih B, Crnkovic D & Karlovic D. Vitamin D and neurotrophin levels and their impact on the symptoms of schizophrenia. *Neuropsychobiology* 2019; **79**: 179-185.

6 Arranz B, Sanchez-Autet M, San L, Safont G, Fuente-Tomas L, Hernandez C *et al.* Are plasma 25-hydroxyvitamin D and retinol levels and one-carbon metabolism related to metabolic syndrome in patients with a severe mental disorder? *Psychiatry Res.* 2019; **273**: 22-29.

7 Zoghbi M, Haddad C, Hallit S, Nabout R, Medlej-Hashim M, Hachem D *et al.* Cognition and physical functioning in patients with schizophrenia: any role for vitamin D? *Nutr. Neurosci.* 2019 e-pub ahead of print Feb 17:1-9. doi: 10.1080/1028415X.2019.1580830.: 1-9.

8 Fond G, Faugere M, Faget-Agius C, Cermolacce M, Richieri R, Boyer L *et al.* Hypovitaminosis D is associated with negative symptoms, suicide risk, agoraphobia, impaired functional remission, and antidepressant consumption in schizophrenia. *Eur. Arch. Psychiatry Clin. Neurosci.* 2019; **269**: 879-886.

9 Ikonen H, Palaniswamy S, Nordstrom T, Jarvelin MR, Herzig KH, Jaaskelainen E *et al.* Vitamin D status and correlates of low vitamin D in schizophrenia, other psychoses and non-psychotic depression - The Northern Finland Birth Cohort 1966 study. *Psychiatry Res.* 2019; **279**: 186-194

10 Coentre R & Canelas da Silva I. Symptomatic correlates of vitamin D deficiency in first-episode psychosis. *Psychiatry J* 2019; **2019**: 7839287.

11 Gurholt TP, Nerhus M, Osnes K, Berg AO, Andreassen OA, Melle I *et al.* Hippocampus volume reduction in psychosis spectrum could be ameliorated by vitamin D. *Schizophr. Res.* 2018; **199**: 433-435.

12 Patel D & Minajagi M. Prevalence of vitamin D deficiency in adult patients admitted to a psychiatric hospital. *BJPsych Bull* 2018; **42**: 123-126.

13 Bruins J, Jorg F, van den Heuvel ER, Bartels-Velthuis AA, Corpeleijn E, Muskiet FAJ *et al.* The relation of vitamin D, metabolic risk and negative symptom severity in people with psychotic disorders. *Schizophr. Res.* 2018; **195**: 513-518.

14 Kulaksizoglu S & B K. The relationship between metabolic syndrome, BDNF,and vitamin D in patients with schizophrenia. *Clinical Neurochemistry* 2017; **11**: 104-111.

15 Akinlade KS, Olaniyan OA, Lasebikan VO & Rahamon SK. Vitamin D levels in different severity groups of schizophrenia. *Front Psychiatry* 2017; **8**: 105.

16 Dogan Bulut S, Bulut S, Gorkem Atalan D, Berkol T, Gurcay E, Turker T *et al.* The relationship between symptom severity and low vitamin D levels in patients with schizophrenia. *PLoS One* 2016; **11**: e0165284.

17 Endres D, Dersch R, Stich O, Buchwald A, Perlov E, Feige B *et al.* Vitamin D seficiency in adult patients with schizophreniform and Autism spectrum syndromes: A one-year cohort study at a German Tertiary Care Hospital. *Front Psychiatry* 2016; **7**: 168.

18 Lally J, Gardner-Sood P, Firdosi M, Iyegbe C, Stubbs B, Greenwood K *et al.* Clinical correlates of vitamin D deficiency in established psychosis. *BMC Psychiatry* 2016; **16**: 76.

19 Bazzano AN, Littrell L, Lambert S & Roi C. Factors associated with vitamin D status of low-income, hospitalized psychiatric patients: results of a retrospective study. *Neuropsychiatr. Dis. Treat.* 2016; **12**: 2973-2980.

20 Bogers J, Bostoen T & Broekman TG. Low levels of vitamin D poorly responsive to daylight exposure in patients with therapy-resistant schizophrenia. *Nord J Psychiatry* 2016; **70**: 262-266.

21 Shivakumar V, Kalmady SV, Amaresha AC, Jose D, Narayanaswamy JC, Agarwal SM *et al.* Serum vitamin D and hippocampal gray matter volume in schizophrenia. *Psychiatry Res.* 2015; **233**: 175-179.

22 Nerhus M, Berg AO, Dahl SR, Holvik K, Gardsjord ES, Weibell MA *et al.* Vitamin D status in psychotic disorder patients and healthy controls--The influence of ethnic background. *Psychiatry Res.* 2015; **230**: 616-621.

23 Zhu DM, Liu Y, Zhang AG, Chu ZX, Wu Q, Li H *et al.* High levels of vitamin D in relation to reduced risk of schizophrenia with elevated C-reactive protein. *Psychiatry Res.* 2015; **228**: 565-570.

24 Yuksel RN, Altunsoy N, Tikir B, Cingi Kuluk M, Unal K, Goka S *et al.* Correlation between total vitamin D levels and psychotic psychopathology in patients with schizophrenia: therapeutic implications for add-on vitamin D augmentation. *Ther Adv Psychopharmacol* 2014; **4**: 268-275.

25 Clelland JD, Read LL, Drouet V, Kaon A, Kelly A, Duff KE *et al.* Vitamin D insufficiency and schizophrenia risk: evaluation of hyperprolinemia as a mediator of association. *Schizophr. Res.* 2014; **156**: 15-22.

26 Cieslak K, Feingold J, Antonius D, Walsh-Messinger J, Dracxler R, Rosedale M *et al.* Low vitamin D levels predict clinical features of schizophrenia. *Schizophr. Res.* 2014; **159**: 543-545.

27 Itzhaky D, Amital D, Gorden K, Bogomolni A, Arnson Y & Amital H. Low serum vitamin D concentrations in patients with schizophrenia. *Isr. Med. Assoc. J.* 2012; **14**: 88-92.

28 McCue RE, Charles RA, Orendain GC, Joseph MD & Abanishe JO. Vitamin d deficiency among psychiatric inpatients. *Prim. Care Companion CNS Disord.* 2012 10.4088/PCC.11m01230.

29 Abdullah AK, Khan S, Mustafa SF, Qutubuddin AA & Davis CM. Vitamin d status and cardiometabolic risk factors in long-term psychiatric inpatients. *Prim. Care Companion CNS Disord.* 2012; **14**: PCC.11m01221.

30 Menkes DB, Lancaster K, Grant M, Marsh RW, Dean P & du Toit SA. Vitamin D status of psychiatric inpatients in New Zealand's Waikato region. *BMC Psychiatry* 2012; **12**: 68.

31 Murie J, Messow CM & Fitzpatrick B. Feasibility of screening for and treating vitamin D deficiency in forensic psychiatric inpatients. *J. Forensic Leg. Med.* 2012; **19**: 457-464.

32 Doknic M, Maric NP, Britvic D, Pekic S, Damjanovic A, Miljic D *et al.* Bone remodeling, bone mass and weight gain in patients with stabilized schizophrenia in real-life conditions treated with long-acting injectable risperidone. *Neuroendocrinology* 2011; **94**: 246-254.

33 Partti K, Heliovaara M, Impivaara O, Perala J, Saarni SI, Lonnqvist J *et al.* Skeletal status in psychotic disorders: a population-based study. *Psychosom. Med.* 2010; **72**: 933-940.

34 Humble MB, Gustafsson S & Bejerot S. Low serum levels of 25-hydroxyvitamin D (25-OHD) among psychiatric out-patients in Sweden: relations with season, age, ethnic origin and psychiatric diagnosis. *J. Steroid Biochem. Mol. Biol.* 2010; **121**: 467-470.

35 Norelli L, Coates A & Kovasznay B. A comparison of 25-hydroxyvitamin D serum levels in acute and long-stay psychiatric inpatients: A preliminary investigation. *E Spen Eur. E J. Clin. Nutr. Metab.* 2010: e187-e189.

36 Rey-Sanchez P, Lavado-Garcia JM, Canal-Macias ML, Gomez-Zubeldia MA, Roncero-Martin R & Pedrera-Zamorano JD. Ultrasound bone mass in schizophrenic patients on antipsychotic therapy. *Hum Psychopharmacol* 2009; **24**: 49-54.

37 Bergemann N, Parzer P, Mundt C & Auler B. High bone turnover but normal bone mineral density in women suffering from schizophrenia. *Psychol. Med.* 2008; **38**: 1195-1201.

38 Schneider B, Weber B, Frensch A, Stein J & Fritz J. Vitamin D in schizophrenia, major depression and alcoholism. *J Neural Transm (Vienna)* 2000; **107**: 839-842.

39 Higuchi T, Komoda T, Sugishita M, Yamazaki J, Miura M, Sakagishi Y *et al.* Certain neuroleptics reduce bone mineralization in schizophrenic patients. *Neuropsychobiology* 1987; **18**: 185-188.

40 Lally J, Ajnakina O, Singh N, Gardner-Sood P, Stubbs B, Stringer D *et al.* Vitamin D and clinical symptoms in First Episode Psychosis (FEP): A prospective cohort study. *Schizophr. Res.* 2019; **204**: 381-388.

41 Salavert J, Grados D, Ramiro N, Carrion MI, Fadeuilhe C, Palma F *et al.* Association Between Vitamin D Status and Schizophrenia: A First Psychotic Episode Study. *J. Nerv. Ment. Dis.* 2017; **205**: 409-412.

42 Yee JY, See YM, Abdul Rashid NA, Neelamekam S & Lee J. Association between serum levels of bioavailable vitamin D and negative symptoms in first-episode psychosis. *Psychiatry Res.* 2016; **243**: 390-394.

43 Graham KA, Keefe RS, Lieberman JA, Calikoglu AS, Lansing KM & Perkins DO. Relationship of low vitamin D status with positive, negative and cognitive symptom domains in people with first-episode schizophrenia. *Early Interv Psychiatry* 2015; **9**: 397-405.

44 Crews M, Lally J, Gardner-Sood P, Howes O, Bonaccorso S, Smith S *et al.* Vitamin D deficiency in first episode psychosis: a case-control study. *Schizophr. Res.* 2013; **150**: 533-537.

45 Dealberto MJ. Clinical symptoms of psychotic episodes and 25-hydroxy vitamin D serum levels in black first-generation immigrants. *Acta Psychiatr. Scand.* 2013; **128**: 475-487.

46 Ghaderi A, Rasouli-Azad M, Vahed N, Banafshe HR, Soleimani A, Omidi A *et al.* Clinical and metabolic responses to crocin in patients under methadone maintenance treatment: A randomized clinical trial. *Phytother. Res.* 2019; **33**: 2714-2725.

47 Fond G, Godin O, Schurhoff F, Berna F, Bulzacka E, Andrianarisoa M *et al.* Hypovitaminosis D is associated with depression and anxiety in schizophrenia: Results from the national FACE-SZ cohort. *Psychiatry Res.* 2018; **270**: 104-110.

48 Krivoy A, Onn R, Vilner Y, Hochman E, Weizman S, Paz A *et al.* Vitamin D supplementation in chronic schizophrenia patients treated with clozapine: A randomized, double-Blind, placebo-controlled clinical trial. *EBioMedicine* 2017; **26**: 138-145.

49 Sheikhmoonesi F, Zarghami M, Mamashli S, Yazdani Charati J, Hamzehpour R, Fattahi S *et al.* Effectiveness of vitamin D supplement therapy in chronic stable schizophrenic male patients: A randomized controlled trial. *Iran J Pharm Res* 2016; **15**: 941-950.

50 Thakurathi N, Stock S, Oppenheim CE, Borba CP, Vincenzi B, Seidman LJ *et al.* Open-label pilot study on vitamin D(3) supplementation for antipsychotic-associated metabolic anomalies. *Int. Clin. Psychopharmacol.* 2013; **28**: 275-282.

51 Tiangga E, Gowda A & Dent J. Vitamin D deficiency in psychiatric in-patients and treatment with daily supplements of calcium and ergocalciferol. *BJPsych Bulletin* 2008; **32**: 390-393.
